# Foreign Psychological Literature

**Published:** 1856-04-01

**Authors:** 


### Part II.

### Foreign Psychological Literature.

### *On the Detection of Doubtful Insanity.*

Our German contemporaries are, at the present time, much occupied with the consideration of doubtful and simulated insanity, and its relations to responsibility, as regards the law and crime. A work, entitled "Reiner Stockhausen," has been published by three physicians—Doctors Böcker, Hertz, and Reichart—which has excited much interest, and which has brought forth a great number of illustrative cases, and many critical comments, not only upon the case, so far as regards the suspected simulant himself, Reiner Stockhausen, but also upon the modes of investigation made use of and the criteria adopted for the solution of the question. The subject is so important that we shall extract freely from the ample details before us, prefacing the account of Reiner Stockhausen by some cases and observations on the subject of simulation generally, by Dr. Snell, director of the Asylum at Eichberg. He observes—

"That the detection of simulation is a very weighty and important task for the physician; for although the general rules to be applied are sufficiently familiar to all, yet in actual practice the difficulties are very great; and he believes that more is to be learned from cases than from general directions.

"Case 1st.—In the house of correction at Eberbach some years ago was a young criminal, who to avoid punishment feigned madness. He worked no more, danced about his cell, sung unconnected words and melodies, and kept up a perpetual humming and growling to himself. He put on a fixed and stupid stare when any one entered his cell, and looked only by stealth at visitors, chiefly fixing his eyes on the wall or ground. To any questions he gave either no answer, or a determinedly incorrect one—thus: How many days are there in the week?—*A.* Ten. When I asked him, Do you know who you are? he answered that he did not know *me*, and had never seen me. On being pressed to say who I was, he said 'A man.' He would not acknowledge to recognise any of his daily associates. The simulation was too plain to admit of doubt; and finding that it availed him nothing, he shortly relinquished it.

"Case 2nd.—Peter U., an unmarried man, twenty-seven years old, was accused of perjury. After he had been two months in prison, he suddenly showed a complete change of conduct. He either would not answer questions, or answered quite astray; he lay apparently asleep on his bed the day long; jumped up suddenly, and ran screaming about the cell; begged to be set at liberty, as 'his dying mother was calling him;' cried 'Fire' when a light was brought, and often pointed to his forehead, and said all was not right there. He was considered insane and sent to this institution."

Here follows a lengthy account of his personal appearance and general conduct, and after that the verbal examination, wherein is manifested the overstrained determination not to approach rationality at any point:—

"*Q.* What is your name?—*A.* *Anton* U. *Q.* Where do you come from?—*A.* Ems. (He lived really in Schlossborn.) *Q.* How old are you?—*A.*

Twenty years. *Q.* Why have you been put in prison?—*A.* Because I stole 2000 florins from Rothschild in Frankfort. *Q.* How did you accomplish it?—*A.* I got in at the widow, and broke the gold chest open. *Q.* With what did you break it?—*A.* With my hands. He did not know the pastor, the schoolmaster, nor the burgomaster of his dwelling-place, nor what number of brothers and sisters he had. He always gave false answers as to what he had eaten, &c. I made him read, write, and count; the two former he did, omitting letters and syllables irregularly; he counted 1, 2, 3, 5, 7, 10, 11, 12, 14, 16, &c.; twice three was seven, twice six fourteen, three times seven sixteen. Being now convinced of his simulation, I tried to induce him to confess it, but every means failed. Nothing remained but to report to the authorities my opinion. It was evident to me that the bewildered and stupid look of the accused made a more powerful impression on the spectators than my scientific conclusions. On the strength of these, however, he was sentenced to two years' hard labour, and on the very day of the judgment ceased his pretence of madness. I visited him afterwards, and found him perfectly sane."

"CASE 3rd.—Katherine R., a widow, bought a house, and afterwards seemed to repent it. In order to set aside the sale, she pretended insanity. With two other *experts* I was appointed to examine into her state of mind. We found a fine old woman, who was partly blind from cataract. Her features expressed indifference, her eyes cast down, but a certain *unrest* not quite concealed thereby. We were told that she could not read or write; she counted 1, 2, 4, 6, 7, 8, 10, 11, 13, 18, &c. How many fingers has each hand?—Four. How much is twice two?—Six. How many children have you?—Nine. (She had seven.) How long has your husband been dead?—Ten years. (It was only five years.) Do you know your daughter here?—what is her name?—Yes; Babette! (It was Katherine.) What year is this?—I don't know. How long is it since Christmas?—I don't know. You have bought a house, have you not?—No; I know nothing of it; I have a house, why should I buy another? Some people wished to buy *my* house. Do you know the Ten Commandments?—what is the first?—I am the Lord thy God. And the second?—I am the Lord thy God. And the third?—I don't know. The same to the fourth. And the fifth?—Thou shalt *not* honour thy father and mother. Convinced of her dissembling, we reported accordingly, though against the testimony of fourteen witnesses, two of them medical. The cause was decided against her, and immediately after that her pretence of insanity ceased. She was committed to the house of correction for perjury."

Dr. Snell adds the following comments on these cases:—

"For the regulation of simulated insanity, it is a favourable circumstance that those who attempt it have ordinarily not the least conception of the true phenomena. They believe that everything about them must be perfectly different from all other men. They must not know any one—must not read, write, or count correctly. On this account it occurs that uneducated people seldom recognise real alienation of mind (unless extreme). Speaking of one in such a condition, they say that 'he cannot be insane; he knows every one, and conducts himself as an understanding man,' and only indicates oddities. They conceive that an evil spirit must enter into all the insane, and change every act and feeling of life. Where they see thought, reflection, and a knowledge of right and wrong, they think that no insanity can exist; whereas *all* these are seldom wanting (at once) in the insane, and they are frequently very highly developed. On this rock most simulants will split, as is seen in the three cases quoted."

Dr. Snell recognises the increased difficulty of detection in cases where an obstinate silence is preserved, and relates an illustrative case, where he still remains in doubt. It requires, however, great self-control permanently to resist all inducements to speak. Two points are dwelt upon as especially worthy of attention.

1. As at the *beginning* of insanity, there is most frequently great disorder of the *emotional* faculties as well as the purely intellectual ones; so if these last appear to be disordered, without any of the former class of symptoms accompanying the onset, the circumstance is *suspicious*.

2. Sleeplessness is perhaps one of the most difficult symptoms permanently to counterfeit. A careful consideration of the physical functions is also absolutely necessary for a correct diagnosis.

We proceed now to the case of Reiner Stockhausen, related by the three above-named physicians. The details are drawn from the work alluded to—from the *Allgemeine Zeitschrift für Psychiatrie* for October 1855 and January 1856, and from the *Correspondenzblatt* for December 1, 1855, and January 31, 1856:—

"R. St. was born near Bonn, about 1797–9. When he was seven years old, his mother married again, and it appears the stepfather was not kind to the boy. St. was of a lively temperament, full of tricks and jests, but could not bear ill-treatment. He soon left his mother's house, and took to a vagabond life. Sometimes working, sometimes idle, he passed through vicissitudes innumerable. On one occasion, he drove post to his native village (Oberwinter), and scattered gold among the people as he passed. Then he made as a common soldier the campaign of 1815. He returned to Neuwied as a captain's servant. He married, and on account of a theft was condemned to three years' punishment. In 1820, he went to America; but after his wife's death at Amsterdam, he returned to Holland, and made his living by hawking crockery, day-labouring, and smuggling. He was again convicted of stealing, and from 1828 to 1832 was condemned to labour at the fortifications. Again, from 1839 to 1850, he was imprisoned in Cologne for theft. When liberated, he drank deep and gambled, and appeared altogether a brutal, degraded wretch. On the 17th of December, 1850, he was again arrested for theft, and gave his name to the magistrate as 'Carl Löwe,' from Bacharach. Again, on the 14th, he gave the same account. Being recognised by another prisoner, and charged with being the often-punished Reiner Stockhausen, he replied, 'I know nothing any more.'

"On the 8th of January examined, his answers were quite astray. *Q.* Do you know Joseph Weber? *A.* I pay no more—now it is done. *Q.* Do you not remember being imprisoned with him in Cologne? *A.* One hundred cost ten groschen, and I don't sell it for less. *Q.* What is your name, and where do you live? *A.* Poniatowski, born at Bernsone, eighty years old.

"January 22.—Gave his name Salentin, answered incoherently in French to some questions, and ultimately gave his name again as 'Carl Löwe.'

"February 4.—To all questions remained obstinately dumb. Dr. Böcker being called upon to give an opinion upon his state of mind, pronounced him a simulant. Between this time and the assizes (June 13) there was a great change of demeanour. He ate very little, and complained much of flying pains in the stomach, chest, and head. He became very filthy in his habits. He answered uniformly to questions, 'It is all gone, all sold; I have nothing more; shoot me dead.' At the assizes, Dr. Böcker and Dr. Hertz were present as witnesses. The former declared that the demeanour of St. was abnormal, but that it was probable he was simulating insanity. The latter suspended his judgment, not having had sufficient time. The case was adjourned to the next assizes, and Dr. Reichart appointed to join his report to that of the other two. We need not quote the examinations at length,—all his answers were quite unconnected and wild. One of the bystanders was one hundred years old; he himself was six days old; his bed was a ship; a small coin was a general's decoration, &c. Afterwards, he became monosyllabic in his replies, but murmured to himself unconnected snatches of his previous answers. He became morose, and tried to kill his keeper; he ate very little and slept very little, and that restlessly; he was very dirty in his habits, but showed no disgust at anything; he witnessed a post-mortem examination without any apparent impression; he was submitted to the influence of chloroform, without any light being thrown on his condition. In September, 1851, the above-named gentlemen gave their reports, separately.

"Dr. Böcker declared that, *apparently*, Stockhausen was not labouring under insanity, and that the symptoms were *probably* simulated. Still less did he suppose that there was any insanity at the time of his last theft.

"Dr. Hertz gave a doubtful and guarded opinion.

"Dr. Reichart gave it as his opinion, that though it might be *possible* that the abnormal mental manifestations were feigned, *apparently* they were not so; that Stockhausen was then deranged, and that at the time of his theft he was not fully sound in mind.

"On account of these diverse judgments, the court decreed that St. should be placed in an asylum, until his state of mind could be more perfectly investigated. He was taken on the 23rd of November, 1851, to Siegburg. Here, according to Dr. Jacobi, he kept perfect silence, speaking on no inducement, or only answering questions by an inarticulate and somewhat fearful growling. He refused all nourishment, except a little bread, and abstained from all employment. He *prowled* about his cell unweariedly, and indulged in many fantastic gestures. After some time, and after the use of the cold douche, he became somewhat more rational; he ceased growling and gesticulating, took his food, and answered questions in the same strain as before. He complained then bitterly of his treatment for a small crime, to which (he said) hunger had driven him.

"He was by degrees brought round, and placed first amongst the *quiet* insane, and then in the convalescent wards, in which he took some share in the ordinary occupations. Ultimately, he appeared as a peaceful, tolerably conversible, intelligent man. He took care of the fowls of the institution for the last few months of his stay there. He became excited greatly, however, so soon as any conversation turned upon the causes of his arrest, or upon his state of mind.

"On the 23rd of November, 1852, when St. had been nearly a year in Siegburg, Dr. Jacobi gave his final opinion that St. was *simulating insanity*, and founded his judgment upon the consideration of the scientific division of insanity into its various forms,—amentia, dementia, idiocy, &c., with none of which Stockhausen's state of mind was found to correspond.

"On the 17th December, St. was brought again before the assizes. His examination lasted twelve hours. On the question of the president, What is your name? he answered, 'You know all things,—you know my name,—you are God.' This answer he returned with little variation to many of the questions. His other answers were equally incoherent.

"Dr. Jacobi and Dr. Reichart gave conflicting evidence as to the mental condition, each stating the grounds upon which such evidence was based. The jury, however, returned a verdict of *Guilty*, and he was condemned to fifteen years' house of correction. The sentence seemed to take little effect upon him, but some days after he told the president, who asked him why he so conducted himself, that he was usually clear in his head, but not always,—that in Siegburg, every one had irritated him greatly; that the douche baths made him desire to put an end to himself, &c. &c. Since this time, he has led a quiet life, as much secluded from all his companions as possible, with occasional outbreaks of violence, but usually peaceable and diligent. The above-named gentlemen have visited him, and had long conversations, which have ended in each being confirmed in his own view. Dr. Hertz is now quite decided on his simulation, Dr. Reichart equally so on his insanity."

Dr. W. Jessen, in his criticism on this work (*Ueber Psychische untersuchungsmethoden, All. Zeitsch. für Psych.* 1855) has some observations upon the method employed respectively by the four examiners to ascertain the state of his mind, which (whether strictly just or not we do not venture to say) are instructive, and from which we may gather these methods themselves. He says:—

"1. The method of Dr. Hertz is ancient, and was till recently the most in use. He sets before himself the question of simulation or real insanity, goes unprejudiced to the inquiry, but only considers the isolated phenomena, and sees in them reasons *for* and reasons *against* simulation. These reasons he compares and weighs, and thereby seeks to determine whether the evidence or counter-evidence be the stronger. This method is bad, inasmuch as the examination of particulars has a tendency to put the *whole* state of mind out of sight and consideration; and this objection becomes ever more weighty, the greater is the acuteness brought to bear upon the examination. Hence the difficulty, and hence the hesitation expressed by Dr. Hertz. This method is justly becoming obsolete.

"2. The method of Dr. Böcker is coming more into use, although it is the worst of all. He also sets before himself the question of simulation, but seems to *pre-suppose* this, and only admits the idea of insanity when this fails in proof. (Many reasons are given at length, for repudiating this method, and much blame thrown upon the trial of experiments, as with chloroform, &c., in such cases,—upon the supposition of simulation.)

"3. The method of Reichart is strictly opposed to the last, inasmuch as he seems to pre-suppose insanity, and only to admit simulation on failure of proof of the former. It partakes of the error of the former method, in being amenable to influence from prejudice, but is a much more just and philanthropic method of inquiry.

"4. The method of Jacobi, in its essential difference from all others, is perhaps not readily comprehended, but is frequently, and perhaps exclusively, used by the highest authorities in psychiatry, as Damerow, Ideler, Jessen, and others. Jacobi does not make the question one of simulation, but founds it upon the condition of the mind generally (Seelenzustand)—viz., whether this be sound or unsound. To decide this, he does not lay especial weight on *any single phenomena*, but upon the *general mental aspect*, which he then compares with the *well-known and recognised forms of mental aberration.* As he believed then, that the condition of mind of St. did not accord with any of these, and as it appeared to him, that all the symptoms were accounted for by the hypothesis of a perverse and neglected character, he declared him *not insane.* We believe, nevertheless, that St.'s state of mind comes under *a certain form* of derangement, and that Jacobi was in error; yet this error did not arise from the method, but from accessory circumstances. One source of error exists in the supposition that the classification of insanity made use of in this case embraced *all possible forms*, which is far from being universally acknowledged. Yet this system, when complete, promises not only to be the most scientifically perfect, but the most practically useful and simple in its application."

This question of simulation is now still further complicated; it is not merely, "Is A. B. of sound or of unsound mind?" but "May he not be of *unsound mind*, and yet dissembling insanity?" In the

*Correspondenzblatt* of January 31, 1856, we find a paper entitled, "Can the insane simulate insanity?" The writer appears to think those in the wrong who, on finding any symptoms of simulation, at once conclude on the absence of mental aberration. We see daily instances of attempts to conceal the mental and bodily condition, and, on the other hand, to feign the existence of pains and sufferings of various kinds.

"The question arises, then, 'Can the insane simulate, not only a bodily, but a mental disturbance?' They can, and they do, if they conceive that the reputation of madness will be of service to them,—and because they, in their insanity, think themselves *not* insane. They place a mask of insanity over the already unsound mind."

The writer concludes his paper by a reference to the case which we have detailed at such length.

"We cannot give a better illustration of our opinion than the case of Reiner Stockhausen, related by Doctors Böcker, Hertz, and Reichart. It is beyond all doubt that St. was a simulant, and therein we agree with Herz and Böcker; but he *was also insane*, and therein we are of the opinion of Reichart. The book is an extremely instructive one, and teaches clearly the important lesson, *that madmen can simulate madness—that simulation and insanity do not necessarily exclude each other, but that they may and do often co-exist.*"

Dr. Damerow, one of the accomplished editors of the *Allgemeine Zeitschrift für Psychiatrie*, has some profound and excellent observations, bearing on this subject, with reference to the neutral ground between sanity and insanity, in the October number of that able journal, p. 643. For the present, however, we must leave the subject.

---

### *Suicide amongst Children.*

(From the *Annales Medico-Psychologiques.*)

M. Durand Fardel states, that amongst 25,760 suicides observed in France from 1835 to 1844, 192 were under sixteen years of age—1 in 134—19 each year. This number seems considerable: our ideas of suicide seem so incompatible with childhood, that we can with difficulty bring ourselves to look upon facts of this nature otherwise than as monstrous exceptions to general rule. The study of them will not be without interest and advantage.

We are left without further particulars of the above-mentioned cases, as to age or circumstance; but we have ourselves collected twenty-six examples of suicide in children from five to fourteen years of age.

One was five years old, two were nine, two were ten, five were eleven, seven were twelve, seven were thirteen, and two were fourteen years old. Seventeen were boys, seven girls, two not mentioned. Amongst twenty-two of them, ten were drowned, ten hung themselves, and two broke the neck. All the girls were drowned. Five of the twenty-six failed in the attempt. Of these last, a woman, mentioned by Esquirol, who had thrown herself into the water at nine years of age, did the same at forty. M. Falret relates the history of a woman affected with suicidal melancholy from the age of twelve years; and of another who, from the age of ten, made frequent attempts at selfdestruction, which succeeded at forty-five. "I know at this time," says Gall, "a young lady, well educated and well brought up, who, at the age of four or six years, when shut up as a punishment, tried to kill herself. She is always awaiting death. She considers it a misfortune to have relatives or friends, since death must soon separate them." We learn from these examples, that however long a time may have elapsed after a first attempt of this nature, there is always a fear of its recurrence. The inadequacy of the motive is often very surprising. One boy of nine years killed himself after having lost a bird; another, of twelve, because he was only the twelfth in his class at school. Sometimes the reasons are more serious. A boy of fourteen years was accused of having stolen a snare for birds, and threatened with imprisonment. He continued to work for a few days, and then hung himself. A child of eleven years old drowned himself, because his mother committed suicide.

In many cases of childish suicide, it appears to be in consequence of punishment or ill-treatment. A child of thirteen years, only son of parents in easy circumstances, was reprimanded and struck by his father. The next day he went to see his companions, and said—"I have been struck by my father: he will not do so again; I am going to drown myself." They laughed at this, supposing it to be a joke; but he effected his purpose, and was found twenty-four hours after drowned. A little girl, eleven years old, was promised by her father a reward if she executed her task well, but threatened with a severe punishment if the contrary. She left home early in the morning, and walked to the quay of St. Bernard. There she met a neighbour of her father's, who asked her where she was going; to which she replied, she was on an errand. All at once she threw herself into the river, but was rescued, after a determined effort to get under a boat.

A child of five years old threw himself into the Elbe, on account of his mother's ill-treatment of him. The suicides of children are almost always remarkable for their *sang-froid* and premeditation. It is certain that before puberty, the idea of death is not accompanied by that sentiment of horror which often, in more advanced life, preserves from suicide. Up to a certain age, children do not comprehend death; somewhat later, they scarcely feel the horror of it. We have seen many children die who were old enough to know that they were about to quit this life; yet we have never observed any expression of terror or despair. On the 7th of March, 1836, Henri Fournier, æt. 12, was sent by his mother for a watch, which he broke. He was sent to bed at 6 P.M. with a piece of dry bread. At 10 o'clock his little sister was sent to see if he was asleep; she returned with the answer that he was. At 6 o'clock the next morning a woman entered his chamber and found him hung. He had made a rope of two cravats, and hung himself to a nail in the wall, climbing up by a wardrobe. Every one bore testimony to his mildness and intelligence; he never complained of ill-treatment, except by once observing that he go punished whilst his sister was always pardoned.

The next case appears to have been without motive, so far as could be ascertained, further than that the boy's uncle had committed suicide a month before under circumstances very similar. Is it an instance of that peculiar spontaneous impulse, called suicidal monomania? or is it merely the result of that powerful principle, imitation?

Benjamin Ricard, æt. 11, was sent by his father to gather cherries, instead of which he returned home, where he met his sister at the door, and told her he had come for a bottle for his father; she replied she had just broken the key in the lock, and did not know how to get in. He then got a ladder and mounted up to his father's chamber. After some unimportant events, he left the house, but soon returned, and having got quit of his sister, he *traced some crosses on the wall*, and wrote on the window-sill with charcoal, "o sadieu de Francois Benjamin Ricard, qui s'est pendu atacher au rido de sa mère." He then hung himself in his bed-room, where was found also a *bottle of holy water*. These circumstances are particularly noticed, because the uncle had before his suicide traced three crosses on the wall, and placed near him a bottle of holy water.

Some years ago, there occurred in the arrondissement of Montargis a double event, as inexplicable as the last related. Pierre Chaumeron, æt. 11, had been playing, on the 2nd of July, 1847, with two children of his own age, as cheerfully as usual. On their returning home, the two said they would call for him shortly to take a walk. In about a quarter of an hour they did so, but he was found hung to a nail in an outer wall, quite dead. It was impossible to account for this act by any past circumstances. He had never suffered any annoyance; he had been in his usual spirits and habits up to the time of the act. It is clearly ascertained that he had no vicious habits—no precocity of temperament. The cause of this suicide remained a mystery. A boy of the same district, aged 14½ years, had followed Pierre to the grave, as a chorister. During the ceremony he was heard to say, "I must hang myself also," and it was laughed at as a jest, though an inopportune one. Four days after the death of his young companion—he had been absent about a quarter of an hour from his parents,—his father wishing to speak to him, sought and called for him, and ultimately found him hung to a nail in the wall, in a precisely similar place and position to that in the last case. There was no assignable cause for such a deed: it would seem to arise from the impulsive necessity *to reproduce in his own suicide* the circumstances which had so much affected his imagination in that of his friend. M. Durand Fardel dwells, after the detail of these cases, at considerable length upon some of the causes which may influence suicide in children; amongst which he ranks chiefly punishments, ill-treatment, and defective education. On the latter subject he urges especially a system of intelligent, and not mill-like, training; showing how necessary it is not only to *educe* those faculties which are inactive, but also to attempt judiciously to repress those which, by an abnormal vivacity, are likely to lead their possessor into evil. After commenting upon the production of suicide by punishment, he proceeds—

We find, in the *Comptes Géneraux*, that in the space of nine years, from 1836 to 1844, there were in France 132 suicides attributed to ill-treatment by parents. Special observations show that these cases occur in all ranks of society. Children are everywhere the same in their affections and impulses; difference of condition has not yet had time to work the specific changes. Everywhere children are found who cannot support brutality, injustice, or even the absence of tenderness. That children but rarely commit suicide is not for want of courage, but that the idea of death does not readily occur at this age.

"But," asks M. Fardel, "is there not a suicide a hundred-fold more sad than that which merely cuts off a life yet new? Children, in whom an evil system of education has once developed this spirit of resistance, this their only protest against the blind authority which oppresses them—these children are almost always ruined for the future destined for them. Whether the intelligence is arrested, or whether the tendency is to crime, all the instincts of oppressed feebleness develop themselves at the expense of the more generous impulses; and they afford to us examples of useless and vicious characters, which, under a more intelligent training, might have been the ornaments of society."

---

*The Theory of Automatism, with the Manuscript of a Monomaniac.* By M. Baillarger, Physician to the Salpêtrière.

(From the *Annales Medico-Psychologiques.*)

"It is more than ten years since I propounded for delirium in general, and hallucinations in particular, the theory of automatism. The more I observe the insane, the more I am convinced that it is in the *involuntary* exercise of the faculties that we must seek the 'point de départ' of all delirium. As soon as cerebral excitement comes on, the subject of it becomes incapable of directing his ideas—the ideas *impose themselves,* and he is forced to *submit* to them. Drawn onward every moment by these spontaneous and involuntary ideas, he ceases to be able to *fix* his attention on anything. After vainly striving against this power which oppresses him, he generally comes to wrong conclusions; for instance, he attributes the suggestions which besiege him to another being. What adds to this belief is, that these ideas are often totally opposed to all which he has had in his sound state.

"We know that nothing is more frequent than conversations in dreams, and it is not surprising that there should be the same antagonism of ideas when the exercise of the faculties has become *involuntary* in the waking state. The resemblance is rendered more complete by the thought often appearing to be articulated internally. Hence sometimes the singular illusion of a voice in the chest or stomach. Their *duality* becomes distinct; there is double thought, and, as it were, two distinct natures."

The writer of the MS. which M. Baillarger gives in illustration of automatism, was a young man who, by severe study commenced rather late, and by onanism, combined with a naturally feeble and irritable constitution, had reduced himself to the deplorable mental condition set forth by himself.

The earlier pages of the MS. describe the more ordinary experiences of an extremely nervous temperament—shyness, uneasiness, and dread of all sorts of dangers. The first indication of *duality* is perhaps to be found in this expression—"I often used to see myself dead, and assisted in imagination at my own burial." The sight of a corpse produced a violent effect upon him, always preventing him from sleeping.

"During the time of which I speak (from sixteen to twenty-seven years of age), I never went to rest without thinking of death—often convinced that I

should die during the night. I foresaw a thousand dangers, fearing to go blind, or to break a leg or an arm simply in walking.

"A man in the enjoyment of natural thought occupies himself with many different and new ideas; he reads, and retains the sense or general impression of what he has read. If any idea does not suit him, he dismisses it, &c., but I cannot do so; I cannot rid myself of my constant ideas—death, the cemetery, the grave, God, and religious ideas."

The following is a remarkable passage:—

"Deprived of natural thought, and of intelligent reflection—if I may so express it—I cannot have any consecutive idea; I cannot occupy myself with any given subject; I am not capable of a moment's attention. *It is matter which has always thought within me.*"

"What (says M. Baillarger) is all this but the automatism of dreaming transported into the waking state?" He further describes himself as incapable of reading, or taking any interest in anything—"c'est comme l'eau qui passe sur la roche de la rivière."

"After passing a day in reading and studying, I asked myself what I had read. I knew nothing; nothing remained with me. One remark, or rather discovery, I have made, which is—that I have never read, like others, with my head; but I have *articulated inwardly* the words, whilst my mind was incessantly occupied with a thousand irrelevant matters." "In attempting to recal a letter which I have writen, I must *articulate* word for word the phrases I have used, or the ideas themselves will not return." At last this process becomes necessary in conversation. "My thought, before being expressed in words, is *formularised* internally; do you understand that?" Again: "There is in my chest or stomach as it were a tongue, which articulates internally. Ordinarily when one wishes to write a letter, the head thinks, the intellect is at work; but with me, the head is of no use: it is the stomach which is at work; c'est cette langue intérieure *qui formule.* I regret not to be able better to explain this sensation."

The next passage we present without translation:—

"Privé de cette pensée instinctive et naturelle à tout homme, je ne puis livrer mon esprit à aucune occupation, je ne suis capable d'aucune attention, ma pensée a changé de siége; tout n'est que matière chez moi, *c'est la matière qui pense.* Ma pensée gît, il me semble, à la poitrine, à l'estomac; je suis portée à croire qu'il y a chez moi *une double pensée,* car il s'opère en moi comme un *contrôle;* il y a comme un autre moi-même qui inspecte toutes mes actions, toutes mes paroles, comme un *écho* qui redit tout, et me représente constamment tout ce que je fais ou tout ce que je dis."

It remains (says M. Baillarger) to indicate the explanation which the patient gives of these phenomena. Instead of seeing in all which he felt a state of illness, he reproaches himself with having by his own fault deviated from the right way. "Little by little I discovered the frightful truth. My existence divides itself into two parts. I lived an ordinary life up to my seventeenth year; but at this epoch I went out of the place assigned me by nature. I followed a false path; j'ai compromis tout mon être moral."

To prove more fully that he has lost his intellectual faculties, and that their functions are performed by the stomach, he says:—

"The proof that I have no *intellectual* thought is, that after having read or written a long time, my mind has never felt fatigued. I have been obliged to cease because my back pained me; the body, matter, demanded repose: but

ISSUE

the mind, thought, never." "A last proof of the same position is this, that if I possessed intellectual faculties I should go mad. A long time ago, when I was, or believed myself to be, in life, I had the idea, without any reason for it, that I should become insane. Now I know, that being deprived of intellectual faculties, I cannot become so."

M. Baillarger concludes thus :—

"The patient had delirious ideas besides those of a hypochondriacal nature. He attributes to himself all unfortunate events; amongst others, the earth quake in 1839. He believed that he could never die, and that the most virulent poisons would be harmless to him. He imagined that every one was examining him, and speaking of him; he therefore avoided society. His affection was, in my opinion, hypochondriacal monomania, brought on by seminal discharges, by a debilitating regimen, and by severe study."

---

### *Practical Observations on the Domain of Psychiatry, with a Retrospect of the last Thirty Years.* By Dr. Steinthal.

(From the *Allegemeine Zeitschrift*, 1855.)

Dr. Steinthal commences by some general observations upon the increased attention bestowed upon mental affections on the Continent during late years, both by the profession and the public; the interest of the latter being evidenced by the vast increase of establishments both public and private for the care and treatment of the insane. Amongst those who by diligence, science, and a philosophic spirit have greatly contributed to the advancement of psychology, he mentions the names of Nasse, Jacobi, Jessen, Flemming, Ideler, Damerow, Bergmann, and many others. Still much remains to be done, and in particular, it is requisite to have a clearer definition of terms, as it appears that the specific forms of insanity have different meanings attached by different writers. Dr. S. considers the classification of Pinel, Reid, and Horn to be the most practically useful—*Mania*, Melancholy, Folly (*i.e.*, general perversion and weakness of the mental powers), and lastly, Fatuity (Blödsinn, *i.e.*, abnormal asthenia of the intellect, palsy of the judgment).

If we enter more closely into the inner nature of insanity, it is recognised as an abnormal condition of Thought, of Feeling, and of Will. "Let us ask ourselves," says Dr. Jessen, "whereby we know that a man is insane. We must answer, we know that he speaks, behaves, and acts otherwise than we have known him to do; and as there is no other manifestation of the psychical life than through speech, behaviour (or disposition), and action, so these being abnormal we consider their source morbidly affected." On this Dr. Steinthal remarks, that however difficult it may be in some given case to distinguish between the somatic affection (mere hypochondria it may be) and the hypochondriacal melancholia already amounting to insanity, or between the mere moral defect and the actual mental aberration, yet the criteria in Dr. Jessen's simple exposition are clearly pointed out, by which we may distinguish insanity from all other diseases. Hence it is important not to found our judgment merely upon the condition of the patient at the time of examination, but to examine into the minutest details of his previous life and conduct—a point often overlooked in judicial inquiries, and the neglect of which is productive of serious error and injury.

"*Concerning the origin of insanity*, no unanimity has been arrived at. Two hostile camps appear opposed to each other, called, according to their principles, the Somatic and the Psychical. The former party are much the more numerous. The principal supporters of the psychical or moral theory, Heinroth and Ideler, differ from each other only in this: that the former seeks the origin of insanity in general sinfulness and criminality; the latter, in excessive or disproportionate excitability. Amongst the supporters of the somatic theory, some consider that the brain, as the seat of thought and the source of action, is *always* and *exclusively* the seat and origin of insanity; others, that it may be produced by disease and suffering of any other organ. Acknowledging myself as one of this party, I must go a step further, according to my individual experience, and seek the chief source of insanity in affections of the abdominal viscera—acknowledging, however, that the abnormities of thought, feeling, and will have their *dynamic* seat in the brain. The overpowering majority of cases treated by me, induce me to attribute the origin of the affection to disease of the digestive organs, and the genital system in the female, and only in a proportionately small number to diseases of other organs, the brain included. Even in cases where, during life, it was scarcely possible to detect any physical anomaly; and when the most prominent reasons seemed to point to a psychical origin; the result of opening the body after death has announced the somatic source of the malady in a very unexpected manner."

In the remarks on *proximate cause* there is nothing of peculiar interest. Hereditary predisposition is mentioned as the most important,—and as an illustration, the writer mentions that in one family he himself treated ten cases, and that four or five others were related to him. Under the head Prognosis, Dr. S., after alluding to the difference betwen public and private treatment, especially urges the necessity of *never, under any circumstances*, relinquishing the hope of cure. He considers, as to treatment, that there has been no advance, but rather the contrary, during the last thirty years—at least so far as medicine is concerned. He likewise enters a protest against the utter exclusion of all methods of coercion; and states, that in his hands, the happiest results have occasionally followed the judicious use of the strait jacket and the rotatory chair. On moral treatment, he says:—

"Decidedly I differ from those who would cure the really insane by so-called *rational* appeals, through religious representations, or methodistical religious practices. Scarcely a single case is known to me, where an evident insanity was checked in the bud by reason, by religious appeal, or by any direct psychical influence. It is quite different when the patient approaches convalescence."

He disapproves *in toto* of the water-cure for insanity—it only produces mischief.

Much stress is laid upon the direct and indirect somatic treatment, and upon the absolute and negative psychical influences. The general observations are concluded by a brief notice of quinine and opium as curative agents. The author has not generally found much benefit from them. The cases mentioned by Dr. S. are extremely interesting, but too lengthy for quotation. They are chiefly directed to the illustration of the somatic origin of affections of the mind, and in particular to the important part which the diseases of the abdominal organs play in the production of aberration. The first is especially instructive, being the case of an old woman (sixty-two), who without any particular bodily affection, *i.e.*, without any defined illness, became affected with melancholia passing into mania, apparently owing to mental trouble, grief, two unhappy marriages, and the like. There was no very especial bodily derangement accompanying the insanity—it seemed a clear case of aberration, dependent purely on psychical causes—yet after death scarcely an organ in the body was found in a normal condition. It might be objected that these morbid changes were the cause of *death*, not of insanity; but most of them were evidently of long standing.

---

### *On the Causes of Insanity.* By M. Trelat.

(From the *Annales Medico-Psychologiques*, 1855.)

M. Trelat comments strongly upon the danger of concluding hastily upon the causes of insanity:—

"A fact exists—we wish to know the cause. Why not take one, almost by chance, in the immense ocean of deceptions, of troubles, of torments of every kind, which agitate humanity! It is always easy to accuse *the unknown*. Misery is great, its privations are cruel; violence, ambition, love, envy, cause many and great sufferings! Is there not sufficient here to account for loss of reason?

"We are not of this opinion; and although we occasionally recognise the influence of an unexpected disaster, of a great misfortune, a violent fear, a disappointment in love; of transports of jealousy, of an excessive abuse of the intellectual powers; we do not hesitate to consider madness from these causes as very rare exceptions.

"Man has been placed in the world with a necessity for labour, with its trials and sorrows; he has received appropriate armour,—vigour, patience, and courage. Unhappiness is rife everywhere—it is the history of man. Strife, more or less painful, is the condition of his existence. Great and prolonged sufferings give a marked superiority to those who endure them. There is nothing more feeble and inefficient than the man who has never suffered. The most persistent adversity has its limits; courage has *none*.

"We believe much more in *internal* causes than in those which are *without*."

#### Illustrations.

"Case 1.—S., æt. 36, falls upon his head, feels no inconvenience at the time, but four years later is seized with melancholia, which is attributed to the fall. On inquiry, it is found that there are three insane persons in his family.

"Case 2.—Madame C., æt. 30, made an unhappy marriage, and suffered much sorrow. She showed symptoms of insanity, which rapidly increased. This was attributed by her friends and her medical adviser to her domestic troubles; but the first inquiries elicited the facts that her mother was insane, and her grandmother had been so.

"Case 3.—A young English girl, living in Paris, was forsaken by a young man who had promised her marriage; she became insane. The victim inspired much interest; which, however, diminished, when it became known that long before this, she had taken to excess intoxicating liquors. This tendency was the result of a morbid state; she died consumptive,—*her mother died insane.*

"CASE 4.—Madame R., æt. 40, had passed a life full of care; the death of her husband threw herself and two young children into great sorrow and poverty. She was attacked with epilepsy and great mental derangement. She had an insane sister. Her daughter had many attacks of epilepsy. One of her nearest relatives was epileptic. It is in *herself*, not in her misfortunes, that the root of the evil is to be sought."

Many other, and even more striking cases are adduced to show that the cause of mental derangement is internal and personal, and not adventitious. The writer dwells at some length upon a very interesting series of four cases, females, who were all attacked with insanity, whilst fulfilling the same, or analogous functions, as superintendent of the insane in the prison of St. Lazare.

"This series of facts of the same nature, in the same place, in so short a time, calls forth very serious reflections. Does madness then propagate itself by imitation? Did not their chances progressively become less and less, by seeing the fate of their predecessors? If this be so, nothing can be more perilous to the reason than exercising any functions about the insane. Should not the physicians themselves be as liable to such attacks as the inferior officials?

"Observation does not support these views. Although these four, who seemed by their age, their physical health, and by every indication of a fine moral organization, to promise long and useful services, had been struck in so similar a manner, we must not be led away by the superficial appearance of the facts.

"It was a pure coincidence—all these had had insane members in their families respectively—one, a sister; another, an aunt and a grandmother; the third, her mother; the fourth, many relatives. Two of the four had had previous attacks of insanity."

The circumstances in which these four persons were placed must, however, be considered as exciting causes, although there was a strong original or constitutional tendency.

"In pointing out the hereditary origin of insanity, we pretend to no novelty. Our examples are chosen from those which abound in insidious appearances, in causes sufficiently numerous and satisfying, to turn us from any further search. There is the danger. Distrust the first aspect of facts—if you only wish for probabilities, you may always have an abundant supply. Where is the man, whose life does not include a sum of sorrow, of misfortunes, or of deceptions, sufficient to *explain*, by the ordinary and received rules, any derangement of intelligence? Were the sentiments and intellect of man given to be unemployed? You make man of too little a stature.

"Labour, calamities, torments, persecutions; strife in all its forms—these are not the source of man's misery—they are his riches and the glory of his character. It is not here that is found the germ of deterioration and death—it is the germ of life and of development.

"The imbecile and the idiot, who do not strive—who think, feel, and suffer little—they have a short life. The intelligent and the strong, whose combat is rough, are generally gifted with long life."

